# A disruptive approach to eliminating weapon-grade plutonium – Pu burning in a molten salt fast reactor

**DOI:** 10.1371/journal.pone.0201757

**Published:** 2018-08-02

**Authors:** Bruno Merk, Dzianis Litskevich

**Affiliations:** School of Engineering, University of Liverpool, Liverpool, United Kingdom; Los Alamos National Laboratory, UNITED STATES

## Abstract

The successful implementation of disarmament treaties of the last centuries has led to significant amounts of weapon-grade Plutonium which are currently stored in high security storage facilities. Disposing this Plutonium should be seen as ‘good housekeeping’ avoiding unnecessary costs and the hazards of storing this material indefinitely. In addition, the disarmament is only brought to a successful end when the Plutonium isn’t available for the production of new weapons anymore. We propose a disruptive approach for Plutonium disposition and demonstrate the feasibility in a neutronic study. Burning of weapon-grade Plutonium in a molten salt fast reactor is significantly more efficient than in the studied other reactors, while efficient process design has the potential to reduce the security concerns significantly. The proposed system could turn about 1.25 tons of weapon-grade Plutonium into electric energy worth £ 0.5 to 1 billion/year, depending on the electricity price while avoiding the hassle and eliminating the risk of high security Plutonium storage. In conclusion, burning of the weapon-grade Plutonium resulting from disarmament could be an economically very attractive approach to reduce the nuclear threat.

## Introduction

“New START, signed by the United States and Russia in 2010 and enacted in 2011, reduces the aggregate number of strategic nuclear warheads and launchers on both sides. The Treaty requires both countries to reduce their arsenals by 2018 to 1,550 deployed warheads.” [1] However, with this current reduction of these additional warheads, the work is only started from nuclear point of view. Since, the warheads maybe disassembled and not usable anymore, but “With regard to storage, most U.S. excess plutonium currently is in weapons pits stored inside insulated double containers in bunkers.” [2]. The final challenge is how to deal with the plutonium pits or in other words how to get rid of the Plutonium and care for the disposition of it in an acceptable way. This will be the key point since only this final disposition or elimination of the plutonium will create the verifiable guarantees for the partners. “As the nuclear-weapon states move towards the elimination of nuclear weapons, transparency and verification arrangements will have to be expanded to ensure the irreversibility of nuclear disarmament.”[3] If the disposition or elimination of the plutonium doesn’t happen “These large stocks of plutonium and HEU [Highly Enriched Uranium] could readily be turned back into nuclear weapons, should political circumstances change in the future. Hence, reducing or eliminating these large excess stocks, rather than simply storing them forever, would be an important contribution to achieving deep and irreversible nuclear arms reduction.”[4]

How can disposition work and why should we do it? “From the point of view of the Nuclear Non-proliferation Treaty the management of excess weapon grade nuclear materials should meet the following requirements:

- make them unusable in nuclear weapons thereby providing the irreversibility of the nuclear arms reductions;
- exclude the risks of their theft and trafficking;
- promote the creation of the monitored regime of nuclear weapons and nuclear materials destruction;
- be economically sound. “[5]

National Academy of Sciences (NAS) has led an influential study on plutonium disposition which led to the result that weapon grade material should be transferred in to a status which is comparable to the ‘spent fuel standard’. The idea is that plutonium should be embedded in a waste form which creates a ‘self-protecting’ gamma radiation barrier comparable to the situation of the plutonium in spent fuel. [2] However, this is still a non-permanent solution since the self-protection will disappear with time. The decay of the self-protection level of Pu stored under the ‘spent fuel standard’ is mostly dominated by the radiation level of the fission products with intermediate half live of ~30 years. Consequently, the radiation level drops with time: ~10% after 100 years and ~1% after 200 years. There would be a significantly better opportunity, transforming the Pu to energy by fission which would lead to a really permanent disposal–this will be the core of this proposal.

Currently, the path followed is still the spent fuel standard. “The international experts meeting held in Paris in the fall 1996 has chosen two preferable plutonium disposition methods meeting the requirements of the ‘spent fuel’ standard:

- immobilizing the plutonium with high-level radioactive waste;
- fabrication it into mixed oxide (MOX) fuel and irradiating the material in civil nuclear reactors.” [5]

However, even the importance of the topic is recognized “Fissile material disposition may also serve as a ‘good housekeeping’ purpose, avoiding the costs and the hazards of storing this material indefinitely; this, indeed, appears to be the motivation behind the disposition plans for a significant portion of the Plutonium that Russia and the United States have declared excess to their military needs.” [4], the whole process haven’t made significant progress. The disposition in the US is delayed due to the problems, delays and the cost overrun in the MOX fuel plant at Savannah River Site the alternative option investigated is deep borehole disposition. The Russian approach for the disposition is the reuse in fast reactors, in the BN-800 especially. However, the start-up core of the reactor has been mainly formed out of UOX fuel and the cost analysis for the production of MOX fuel for fast reactors has shown that the MOX fuel production is the main cost driver in the complete closed fuel cycle [6]. In addition, it will be shown later that even with high Pu loading, the plutonium vector is only partly degraded. In the case of an optimized core design the system could even be used to produce more weapon grade plutonium out of the inserted one.

**Fig 1:**
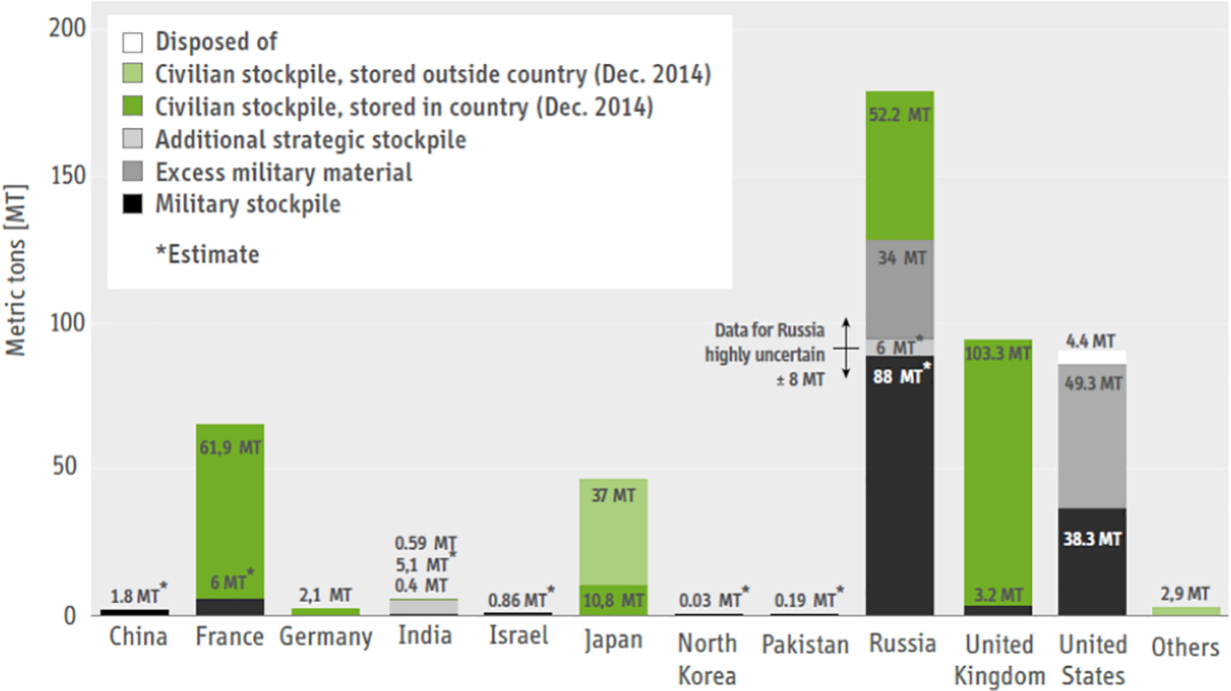
National stocks of separated plutonium as endo of 2014 (military stocks are based on IPFM estimates except for the United States and United Kingdom whose governments have made declarations) [12].

Efficient disposition of weapon grade plutonium is not only a matter of reducing cost for storage and safeguarding, it has to be seen as an important action for counter terrorism since it will significantly reduce or even eliminate the risk of misuse or theft. “In principle, disposition of these large stocks–which means physically transforming them into forms that would be difficult and costly to recover for use in nuclear weapons–could also decrease the risk that some portion of them could be stolen and fall into the hands of terrorists or proliferating states”[4]. It always has to be kept in mind that “separated Pu is a more immediate and urgent issue, because the most difficult task of extracting Pu from the intensively radioactive spent fuel has already been performed; the remaining steps for incorporating the material into a nuclear bomb are much easier.” [7]. Looking into the amounts of excess military materials (see Fig 1) demonstrates the level of the challenge. Especially, if we remind the following figure which always has to be kept in mind, “Only about five kilograms of such plutonium [from dismantled nuclear weapons] is needed to make a primitive nuclear weapon in the kiloton range.”[7]

Collecting all mentioned points, including the delay of all current programs, it seems to be indicated to re-think the currently envisaged approaches to analyze opportunities for more advanced solutions. Ideally providing solutions with a lower life cycle cost which are maybe even completely irreversible, not only make it difficult and costly to recover the plutonium.

Could we do plutonium disposition in a more efficient and economical way in a highly innovative nuclear reactor?

Recently, molten salt reactors have been proposed for highly efficient transmutation and for the burning of civil plutonium [8, 9, 10, 11]. Could this systems be used for the disposition of weapon grade plutonium, too?

Burning of civil Pu is mainly considered with focus either on partitioning and transmutation (P&T) (eliminating Plutonium and ideally minor actinides from the progression into the final disposal to reduce the long term radiotoxicity) where the produced energy is recognized as a nice asset or with the operation of a fast reactor fleet where the Plutonium is considered as an asset (fuel for the reactor fleet which will be recycled) where the aim is to reproduce the plutonium and to keep a reasonable plutonium vector for the next insertion. The main aim of the P&T is the reduction of the radiotoxicity in the final disposal and thus burning of a complex Pu and ideally minor actinide vector, with Pu as major problem due to the significant amount, while avoiding the build-up of higher actinides. It has been demonstrated that a fast reactor spectrum is essential to achieve this aim. In some countries like UK a civil Pu stockpile has been created due to successful reprocessing, but a delay in the steps required to reinsert the plutonium in any kind of reactor.

The major drivers in the burning of weapon grade plutonium are different, mainly creating a Pu vector which hampers a reuse of the Pu for weapons and having a process which avoids opportunities for diverting plutonium. It is to clarify if a molten salt fast reactor which has demonstrated an efficient burning of civil Pu is a good approach regarding the changed requisite, too.

### Why molten salt reactors?

Already 1978, Engel et al. have worked out one of the most attractive features of molten salt reactors. When the plutonium is once inserted into a molten salt reactor system it is not necessary to separate it in any of the steps of the burning operation when the system is designed in the right way [13]. This basic discussion has been taken up 1992 when Uri Gat et al. proposed the MOLTEN SALT REACTORS FOR BURNING DISMANTLED WEAPONS FUEL in a technical note in the ANS Journal Nuclear Technology. They collected really strong arguments supporting molten salt reactors as highly interesting system for the burning of plutonium: “The MSRs have the flexibility to utilize any fissile fuel in continuous operation with no special modifications, as demonstrated in the Molten Salt Reactor Experiment, while maintaining their economy. The MSRs further require a minimum of special fuel preparation and can tolerate denaturing and dilution of the fuel. Fuel shipments can be arbitrarily small, which may reduce the risk of diversion. The MSRs have inherent safety features that make them acceptable and attractive. They can burn a fuel type completely and convert it to other fuels. The MSRs also have the potential for burning the actinides and delivering the waste in an optimal form, thus contributing to the solution of one of the major remaining problems for deployment of nuclear power.” [14]

Especially, the points, no need for separation and the instantaneous mixing of all inserted materials (freshly inserted weapon grade Pu would immediately mix with the reactor grade Pu which is already in the core) create a clear advantage which is in strong contrast to the fuel management like it is used in solid fuelled reactors with the separated steps: reprocessing, fuel production, and fuel irradiation including the therefore required transports required between the sites. A detailed analysis and comparison of general features of liquid metal cooled fast reactors and molten salt reactors regarding the burning of plutonium is given in [9]. Clear operational as well as efficiency and safety advantages of molten salt reactors are worked out, there.

Summarizing the advantages, the following conclusions have been drawn for the burning of reactor grade plutonium in a molten salt fast reactor system which support the use for the burning of weapon grade Pu, too:

- flexibility to utilize fissile fuel in continuous operation
- minimum of special fuel preparation
- significantly reduced fuel shipments
- inherent safety features
- limitation of the weapon grade plutonium availability and reduction of the risk of proliferation
- fissile material which is once inserted is not needed to be taken out anymore [8]

All these points together demonstrate the high attractiveness of the molten salt reactor technology for the application as burner for weapon grade plutonium where proliferation resistance and limitation of access opportunities are of utmost importance. In addition there are some strong arguments for an expected cost reduction in comparison with solid fuelled systems:

- replacing traditional reprocessing with a strongly demand driven salt clean-up
- avoiding of cool down storage and transfers like in solid fuelled systems
- applying low pressure technology
- avoiding solid fuel production; a major cost of the fast reactor fuel cycle

### Development of molten salt reactors

Molten salt reactors have already a long history, based on the curious idea, the nuclear aircraft. A proposal of the early phase of nuclear in the late 40s and early 50s which led to the Aircraft Reactor Experiment, a small thermal reactor experiment with only 2.5MW. [15] This first experiment has followed to a follow up, the Molten Salt Reactor Experiment (MSRE), an 8 MW thermal experimental reactor which has been developed in an impressing short time period. “Design of the MSRE started in the summer of 1960 and construction started 18 month later, at the beginning of 1962. The reactor went critical in June 1965, and was briefly at full power a year later “[15].

In the last years, the molten salt reactor technology has regained interest. Many countries worldwide contribute with different intensity to molten salt reactor (MSR) technology, among which are the European Union, the United States, the Russian Federation, Japan, India and for the past few years China and Korea. In Europe, this is manifested in several EURATOM projects, MOST–review on MOlten Salt reactor Technology [16, 17], EVOL- Evaluation and Viability Of Liquid fuel fast reactor systems [18, 19, 20] and SAMOFAR—Safety Assessment of the Molten Salt Fast Reactor [21] and the Russian Project: “Minor Actinides Recycling in molten salts” (MARS) [22, 23, 24] which has been linked in a Euratom–Rosatom collaboration MARS-EVOL on molten salt reactor. In contrast to the historic MSRE, the focus of the current projects is on the development of a molten salt fast reactor without graphite used for moderation in the MSRE.

The reactor proposals mentioned above operate in a fast neutron spectrum and, thus, would be well suited for the burning of the weapon grade Plutonium. However, there are other technologies which have been proposed earlier like light water reactors (LWRs) with mixed oxide (MOX) fuels and sodium fast reactors with MOX fuels. The different reactor concepts can vary in their efficiency to perform this task significantly. Which is the most efficient system for burning Weapon Grade Plutonium? A comparative analysis of the efficiency of different reactor types to burn WGP (weapon grade plutonium) is given in the following chapters to identify the most attractive approach based on different view points

## Materials & methods

The raw data of the calculations (Aurora, HELIOS and ZENITH files), the used PYTHON script for the cycle calculations and the Excel file for the data acquisition are available under [25].

### Reference configurations

#### Molten salt fast reactor (MSFR)

**Fig 2:**
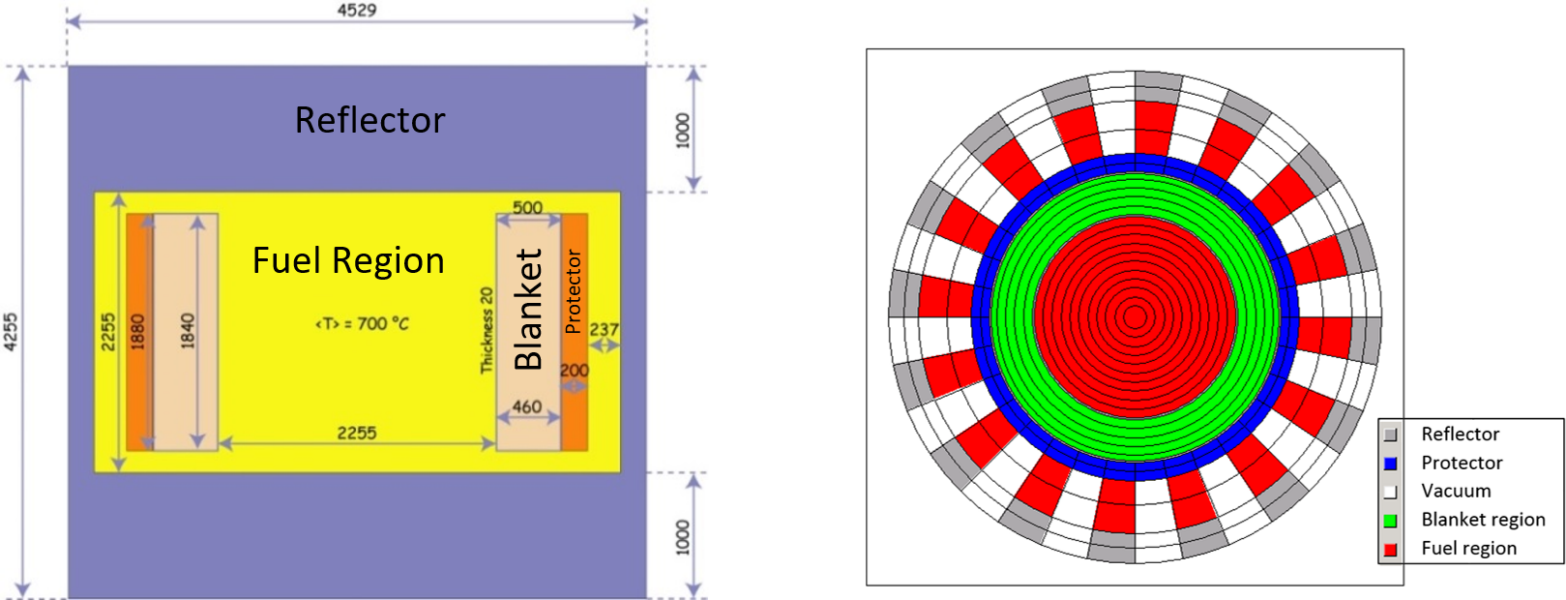
left: EVOL Benchmark definition [27], right: Volume corrected 2D HELIOS model of the molten salt reactor.

The calculation for the MSFR is based on the dimensions and boundary conditions (e. g. average temperatures) given in the EVOL benchmark definition (see Fig 2), but with adopted salt due to the experience with earlier calculations for fertile free systems. The reference system has a 3000 MW_th_ core with fast neutron spectrum formed as a single cylinder. The fission reactions occur in the flowing fuel salt in this cylinder [26]. The dimensions are given in Fig 2. In general, the core is composed of the active core, the lower injection area, the upper extraction area, and the out of core area consisting of the heat exchangers and the pumps.

For the simulations, the HELIOS 2.1 code is used [28] where the benchmark configuration is translated into a volume corrected 2D HELIOS model (see [29]) with a leakage correction available in HELIOS (determined based on the EVOL critical configuration and the material composition of the EVOL benchmark) to reflect the leakage in the third dimension while the leakage in radial direction is directly modelled. The model has been detailed by introducing 16 heat exchanger pipes compared to the EVOL benchmark configuration to improve the leakage estimation.

**Fig 3:**
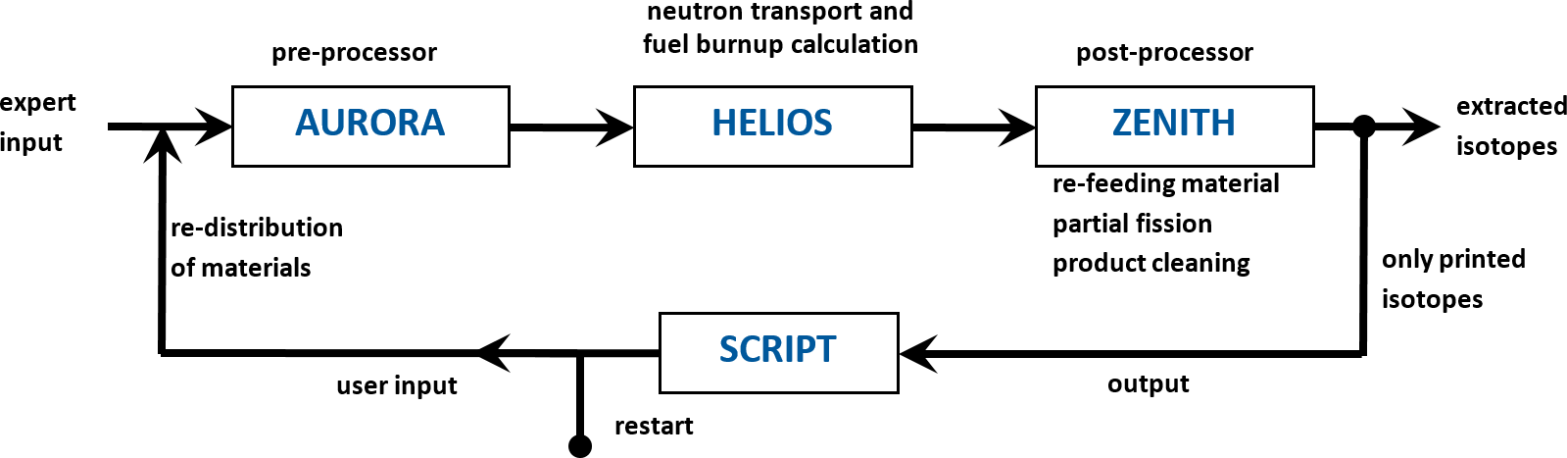
Description of the calculation cycle for the simulation of a MSR.

The used HELIOS code package is a licensing grade code module which performs the neutron transport and burn up calculations, and has been developed for cross section preparation in defined calculation areas. The code has been developed for the simulation of solid fuel structures. Thus, the possibility of online re-fuelling as well as online salt clean-up is not foreseen. To deal with this MSR challenges a PYTHON script has been developed while all basic input data is stored in an expert input, the material configuration is reproduced in every cycle using the script while both inputs are merged in the pre-processor AURORA, see Fig 3. Within each HELIOS cycle 5 burnup steps are calculated. The results of each cycle are evaluated in the post-processor ZENITH where the requested isotopes will be fed back. Theoretically, the selected scheme is able to simulate the processes accurately by using small time steps. Two different time scales are applied for the salt clean-up, one for the helium bubbling for volatile and gaseous fission products as well as the online salt clean-up for the soluble fission products. A detailed description of the modelling is given in [9].

The salt configuration and the corresponding thermodynamic data is taken from the MOSART project [23], 17LiF-58NaF-25BeF_2_ (mole %) mixed with PuF_3_ based on WGP, a salt composition which has been developed for a fertile free system. The heavy metal content in the salt is continuously increasing due to the feeding of weapon grade Plutonium and the formation of higher actinides (~1 t at start to slightly less than 10 t) which leads to a cycle time increasing form ~16 days for the first cycle to ~150 days for the asymptotic cycle.

#### Fast reactor assembly

The fast reactor fuel assembly model is based on the data for the European Fast Reactor. The data is mostly taken from the IAEA Fast Reactor Database—2006 Update [30] with some special data taken from Waltar, Reynolds: Fast Breeder Reactors [31] and the European fast reactor (EFR) fuel element design [32], see Table 1. The input parameters for the simulations were chosen to reproduce the typical design and operational parameters of fast reactor fuel assemblies.

**Table 1:** Basic data for the fast reactor fuel assembly.

Outer pin diameter	8.5 mm
Cladding thickness	0.52 mm
Pitch to diameter	1.2
Can wall thickness	4.5mm
Fuel density	9.26 g/cm^3^

**Fig 4:**
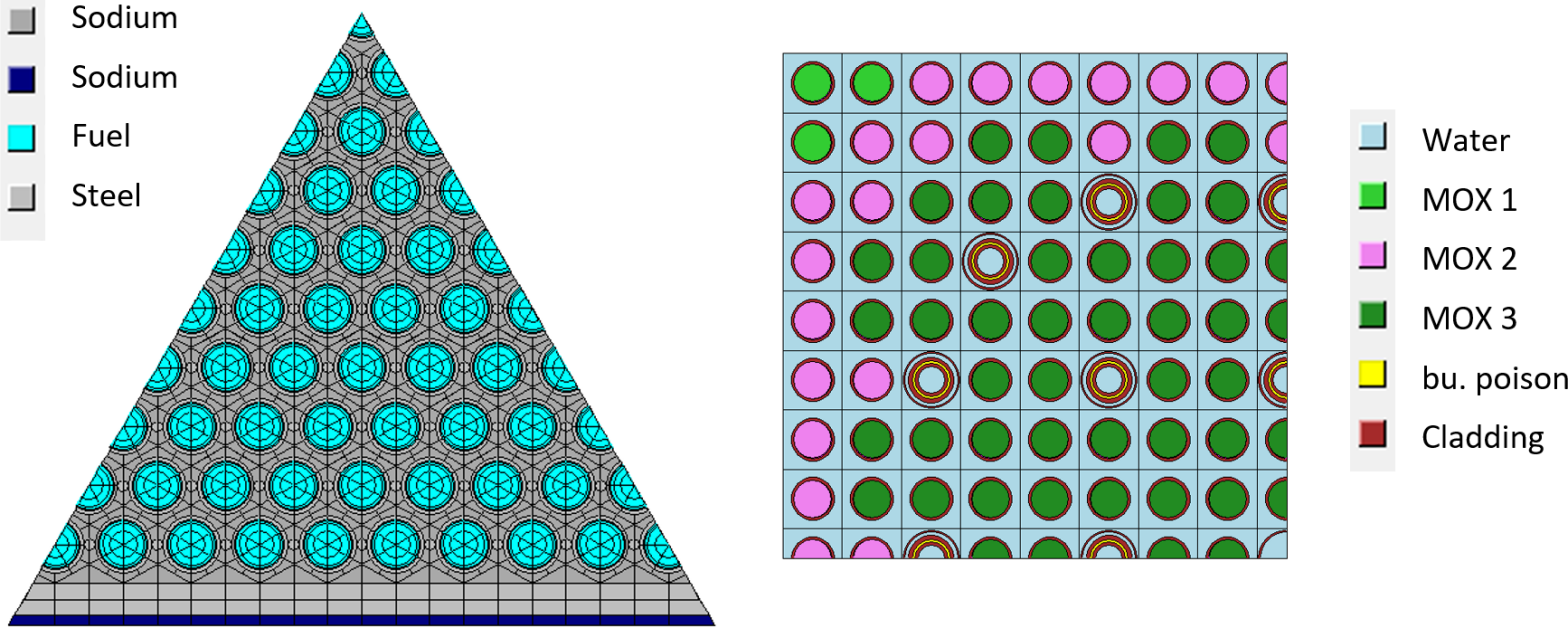
1/6 of the sodium cooled fast reactor fuel assembly based of EFR (left) and 1/4 of the LWR fuel assembly based on the NEA MOX benchmark (right) with wet annular burnable absorbers.

Cladding, spacer wire and can wall consist of SS 304 along the HELIOS definition [29]. The temperatures: cladding 635°C, wrapper and can wall 545°C, and fuel 1100°C. The fuel assembly consists of 10 rings of fuel rods following the EFR design, see Fig 4 (1/6 symmetric part of one fuel assembly). The applied power density is 118.8 W/g corresponding to the maximum power density in the EFR. The reactor core is formed of fuel assemblies with 18.3%, 22.4%, and 26.9% plutonium content in mixed oxide fuel. The maximum burnup has been set to 200 GWd/tHM which has been chosen based on data from [32, 33].

#### LWR MOX fuel assembly

The LWR fuel assembly model is following the OECD/NEA AND U.S. NRC PWR MOX/UO2 CORE TRANSIENT BENCHMARK [34] definition. The MOX assembly consists of three types of pins using 2.5% (MOX 1), 3% (MOX 2) and 5% Pu_fiss_ (MOX 3) content, and wet annular burnable absorber within the guide tubes.

The fuel assembly consists of a 17 by 17 arrangement (see Table 2) with 24 guide tubes, and one instrumentation tube. The HELIOS model is given in Fig 4. The average Pu_fiss_ content in the fuel assembly is 4.3% Pu_fiss_ and the target burnup is 50 GWd/tHM.

**Table 2:** Geometric data of the LWR-MOX fuel assembly.

Outer pin diameter	9.166 mm
Cladding thickness	0.573 mm
Pin pitch	12.6 mm
Assembly pitch	21.42cm
Fuel density	10.41 g/cm^3^

### Calculation tool and material

All comparison calculations are performed using the HELIOS 2.1 code which is a 2D spectral code with wide unstructured mesh capabilities and a transport solver using the collision probability method [35]. Some approximations have to be accepted for the molten salt as well as the fast reactor application. No fuel salt movement can be modelled within the cycle, thus the fuel is only redistributed when a new input is written. The HELIOS package is developed for LWR application and a LWR spectrum is used for the weighting of the master libraries within each energy group. However, comparisons to SERPENT on the isotope accumulation in a fast reactor configuration have shown good agreement for the major isotopes [36]. The approximations and the use of the HELIOS code package seem to be adequate for this kind of comparative investigation simulating different reactors with significantly different neutron spectra.

The used TRU isotopic vector for weapon-grade Pu is given in Table 3.

**Table 3:** Isotopic composition of Pu separated from different reactors about 1975 in comparison with weapon-grade Plutonium defined by US-DOE and US-NRC [37].

Reactor type	Burnup GWd/t_HM_	Plutonium isotopic composition				
		Pu-238	Pu-239	Pu-240	Pu-241	Pu-242
**MAGNOX**	**5**	**~0**	**0.685**	**0.25**	**0.053**	**0.012**
**CANDU**	**7.5**	**~0**	**0.668**	**0.265**	**0.055**	**0.012**
**PWR**	**30**	**0.016**	**0.565**	**0.238**	**0.128**	**0.053**
**Weapon-grade Plutonium (US-DOE, US-NRC**	**very low**	**0.00012**	**0.938**	**0.058**	**0.0035**	**0.00022**

## Results and discussion

### Reactor specifics

The modelling and simulation study for the burning of weapon-grade Pu has been performed applying the described Python script using the HELIOS code system. There are some reactor specific boundary conditions which have to be kept in mind to understand the differences seen in the Pu burning rates given in Table 4. In the solid fuelled reactors, thus in the light water reactor (LWR) as well as in the fast reactor (FR), the fuel will be inserted in fuel assemblies. Fuel assemblies in this kind of reactors are inserted for several operational cycles (~ 3 to 5 years), but only a part of the available fissile material will be burnt during one operational insertion. This is caused by the specifics of the reactor systems where so called excess reactivity is required to operate the reactor, thus the operation relies on having sufficient reactivity reserve which is eaten up during the cycle partly due to the burning of fissile isotopes and partly due to the accumulation of fission products which can act as neutron absorber. However, due to safety reasons, the absolute amount of excess reactivity and thus the length of the operational cycle is limited. At the end of the life of the fuel assembly, there would be the possibility to reprocess the fuel to separate the remaining fissile material to insert it into new fuel assemblies. However, the current reprocessing scheme is built on the separation of fissile material and can be seen as proliferation concern in itself, especially if the Plutonium vector still contains a high amount of Pu-239 like it appears in the sodium cooled fast reactor case. In contrast to this, the proposed molten salt fast reactor (MSFR) is operating in a continuous mode. Here, the first core configuration is just critical (no excess reactivity) and the burnt fissile material is continuously replaced by fissile material which is provided by the online feeding system. A comparable process is proposed for the salt clean-up, a small share of the salt is continuously separated from the core which is foreseen to be chemically cleaned from the fission products which prevent the reactor from long term operation [10, 38, 39]. This proposed highly innovative approach avoids any separation of fissile material and thus leads to a significant reduction of the proliferation and security concerns.

**Table 4:** Isotopic composition of Pu loaded and unloaded from the different studied reactor systems.

	MSFR		LWR		SFR 18.3% Pu	
	load	unload	load	unload	load	unload
Pu-238	0.01%	2.0%	0%	0.7%	0.01%	0.1%
Pu-239	93.82%	40.5%	93.60%	45.4%	93.82%	79.8%
Pu-240	5.80%	43.4%	5.90%	30.5%	5.80%	18.3%
Pu-241	0.35%	7.3%	0.40%	17.8%	0.35%	1.6%
Pu-242	0.02%	6.8%	0.10%	6.3%	0.02%	0.2%
Pu destruction rate		89.67%		36.84%		10.9%

### Isotopic compositions

Based on this different operational approaches, different time schedules appear in the systems. The given Pu burning rate in LWR and FR is achieved when the fuel assembly is extracted from the core, thus after 3 to 5 years. However, if a higher burning rate is expected reprocessing would be required demanding several years of cooling if based on the current aqueous technologies. This step would have to be followed by the costly production of new MOX fuel for the reactor operation [6]. The burning rate is calculated by plutonium at end of operation divided by the inserted amount of Plutonium.

The given burning rate in the MSFR is evaluated at the end of operation of the reactor, in this simulation after 60 years. The burning rate in a MSFR is increasing with every day of operation due to the online feeding system which replaces the burnt Pu share from the initial loading. It could be seen like a basin of water in the sun which have to be kept full. First you have to fill the basin–this is comparable to the initial core loading which has to be filled with sufficient fissile material. When the water in the basin is warmed by the sun a small share of water will slowly evaporate which has to be compensated with a small continuous water make-up system. When the system is operated long enough more water will be evaporated than the content of the basin, but in the evaporation rate you always have to consider the first filling of the basin and that the basin is still full at the end of operation. The same process takes place in the reactor, there is the initial filling, there is the continuous make-up of burnt fissile material, and there is the end of life core (which could be used in a next stage reactor).

Taking the described systematic differences into account it is obvious that the burning rate achievable in MSFR is impressive. In contrast to this, the destruction rate in the solid fuelled system is limited within one stage and would request reprocessing and fuel reproduction before a reinsertion into the reactor. The Pu destruction rate coincides very well with the previous study [8].

The light water reactor burning destroys only a part of the weapon grade plutonium, but it leads to a Pu vector which will raise only reduced proliferation concerns due to the low Pu-239 content and the high Pu-240 content. However, exactly this low content of fissile Pu isotopes (Pu-239 and Pu-241) limits the multiple recycling opportunities in light water reactors. Thus the approach to hamper the weapon use has been successful, but a leftover is produced which is comparable to the problem of civil plutonium. On the one hand, in the SFR case the Pu quality is still very good–high content of Pu-239 –which would allow a sustainable multi-recycling scheme and thus more efficient burning. On the other hand, exactly this good Pu quality will raise concerns regarding proliferation and misuse when the fissile material is recovered during reprocessing. In addition, the solid fuelled systems will provide an advantage in flexibility, single fuel assemblies can be inserted, maybe even into already existing reactors. The efficiency of the LWR application for Plutonium burning is much higher than for the fast reactor, since the breeding of new fissile material (Plutonium) from the Uranium matrix is less efficient in a thermal spectrum. This is why a fast spectrum reactor can act as breeder while this isn’t possible in a thermal light water reactor with mixed oxide fuel. However, as fast reactor could be designed in a way that the burning of Plutonium is more efficient to a certain limit, see e. g. the CAPRA project [33, 40]. However, the problems of the solid fuel arrangement and the request for reprocessing and solid fuel production would not disappear.

Based on the clear advantage of the application of a molten salt reactor for the burning of weapon-grade Plutonium, a more detailed analysis is given in Table 5 and Table 6. The operational data indicates, that the core is started with a comparably small Pu amount of about 1 ton, while more than 74 tons are fed into the system during almost 60 years of operation. For quality assurance of the modelling and simulation, the comparison of the burning rate over power achieved in the simulation with the theoretical achievable burning rate over power calculated based on energy per fission for Pu-239 is given. The almost perfect coincidence of this two values with the uncertainty margin gives trust into the simulation and the following evaluation with an excel sheet. The real burnt rate of Plutonium is based on the full isotopic evaluation, thus all actinide isotopes which are left over are considered, not only the plutonium. This leads to the slightly lower number which is however based on a more detailed dataset.

**Table 5:** Operational data for the molten salt fast reactor case.

initial Pu load	0.97	tons
inserted Pu	74. 1	tons
op. time	59. 8	years
burning rate over power	42.56	kg/TWh
Theoretical burning rate over power	42.58	kg/TWh
Real rate burnt	88.8	%

**Table 6:** Detailed heavy metal isotopic composition of the MSFR core at begin and end of operation.

Isotope identifier	unload vector	load vector	Integral load mass [t]	unload mass [t]
Np-237	0.1%			0.006
Np-238	0.0%			0.000
Pu-238	1.9%	0.0%	0.01	0.158
Pu-239	37.2%	93.8%	70.43	3.137
Pu-240	39.9%	5.8%	4.354	3.364
Pu-241	6.7%	0.4%	0.26	0.565
Pu-242	6.2%	0.0%	0.02	0.527
Am-241	2.2%			0.188
Am-243	1.9%			0.163
Am-242m	0.1%			0.012
Cm-242	0.1%			0.011
Cm-243	0.0%			0.003
Cm-244	2.1%			0.177
Cm-245	0.6%			0.052
Cm-246	0.4%			0.033
Cm-247	0.1%			0.008
Cm-248	0.1%			0.008
Bk-249	0.0%			0.000
Cf-249	0.0%			0.001
Cf-250	0.0%			0.000
Cf-251	0.0%			0.000
Cf-252	0.0%			0.000
U-233	0.0%			0.000
U-234	0.0%			0.000
U-235	0.1%			0.006
U-236	0.2%			0.015
U-237	0.0%			0.000
U-238	0.0%			0.000
	100.0%		75.07	8.43

The detailed analysis of the heavy metal composition (Table 6) indicates, that the end of life (EOL) content of heavy metals is about a factor of 8 higher than the initial heavy metal loading. From the table different information can be extracted:

- The Pu-239 mass is significantly higher than in the initial loading, thus the system requires at EOL significantly more fissile material. This can be explained with the presence of many isotopes which absorb neutrons, some of the fission product isotopes, as well as some of the bred higher isotopes.
- In the long term operation of the reactor a considerable formation of higher isotopes is formed due to absorption processes (breeding). The bred heavy isotopes form about 60% of the EOL heavy metal content.
- The EOL isotopic composition of a molten salt fast reactor operating 60 years on weapon grade Plutonium feed (0/94/6/0/0) to unload (2/41/43/7/7) diverges less than expected from one operating on reactor-grade plutonium feed (3/55/24/11/7) to unload (4/22/46/10/17) case, see [8]. The long term operation has eliminated a major part of the difference in the feed composition
- The final molten salt loading would have to be seen as high level nuclear waste, or it would have to be reused for the operation of a next generation reactor [10]

**Fig 5:**
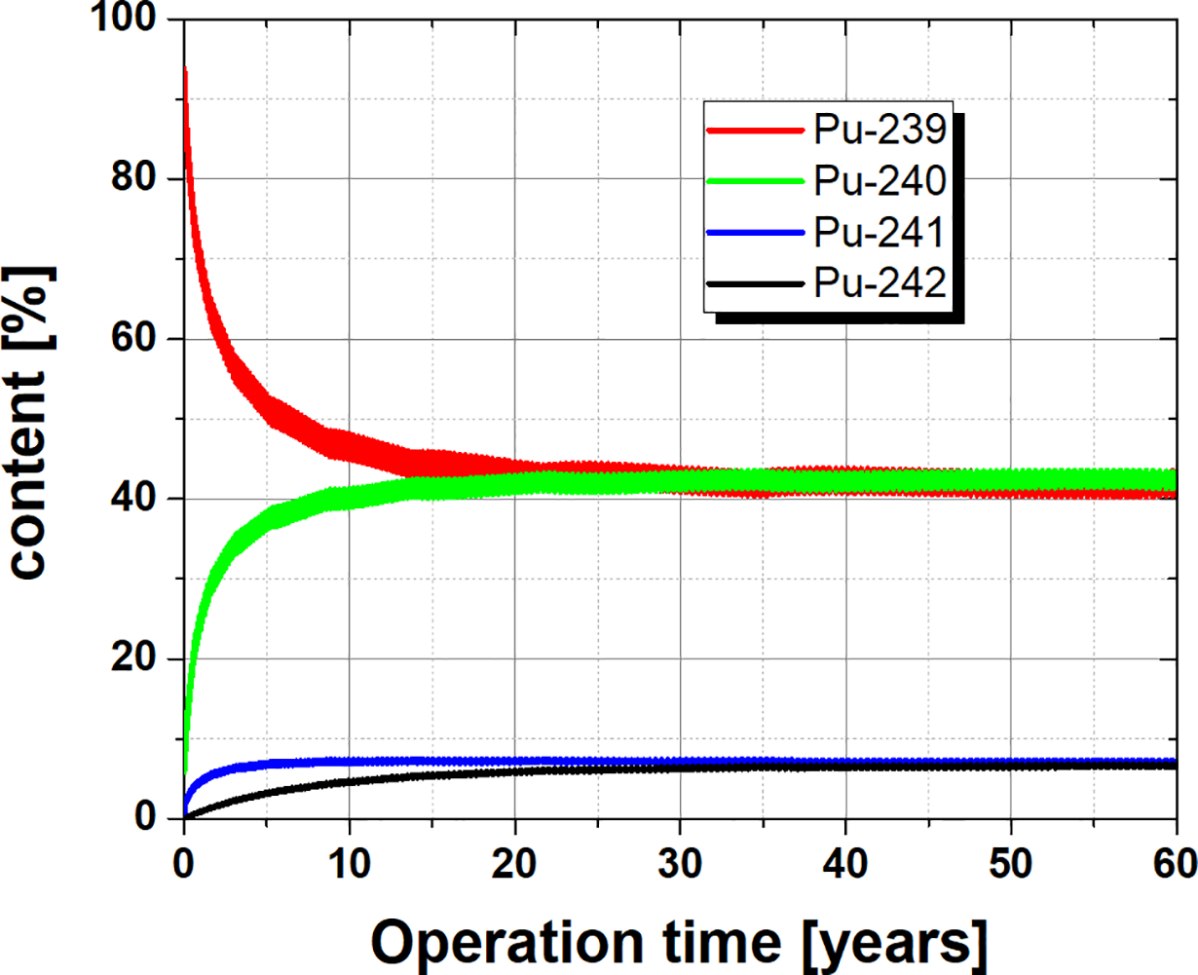
Major contributors to the Plutonium vector over lifetime in a molten salt fast reactor.

However, not only the end of life composition demonstrates the change of the isotopic vector, the operational analysis of the fuel composition, see Fig 5 provides another strong argument. The impressive point is, that already after about 2 years of operation, the Pu-239 share is below 60%. This change is impressive, considering the background of the continuous feeding of weapon grade Plutonium with more than 93% Pu-239. This means after only 2 years of operation the fresh weapon grade plutonium which is inserted is mixed into a matrix which provides a good proliferation resistance and can’t be extracted anymore. Thus the approach of using a molten salt fast reactor provides not only a long term solution by efficiently burning Plutonium, it also provides after a very short start up time a high proliferation resistance and robustness against misuse and theft which is complemented with the integral design which avoids handovers in the fuel cycle.

Another point to be considered is the total radioactivity and radiotoxicity of the material. Burning plutonium eliminates unwanted Pu-239 isotope but depending on the reactor system producing different amounts of heavier plutonium isotopes and transplutonium elements. This can result in increasing total radioactivity and essentially unchanged radiotoxicity of the material with the corresponding load on the society and the environment. In general, fast reactors have significantly higher fission probability for transuranium isotopes (TRUs) than thermal reactors [41], thus fast reactor systems will lead to a reduced build-up of TRUs. Considering the massive reduction of the plutonium amounts and the very limited unloading masses of TRUs the destruction of the weapon grade plutonium will reduce activity as well as toxicity of the whole considered system, too.

### Commercial opportunities

However, the Pu disposition should in our view not only be analyzed and discussed form security point of view. Another important parameter would be the cost, or the economic opportunity given by the separated high level fissile material–at least as long as the use doesn’t produce a significant concern regarding proliferation or misuse. The estimations based on the following data:

60 years of theoretical full power operation corresponding to ~65 years of operation when a revision time of 4 weeks per year is estimated.

- Thermal reactor power 3 GW_th_
- Proposed net efficiency 40% for a high temperature system
- Operational hours per year 8650 h

Using the given data the electrical power is calculated to 1.2 GW_el_. The operational hours will accumulate to 519000 operational hours within 65 years and could lead to 625 million MWh of produced electricity. The long term market price for this electricity is hard to guess, but there are some indicators. The UK has guaranteed EDF a price of £92.50 per megawatt hour (MWh) for the Hickley Point C project and £89.50 if EDF develops another new reactor in Sizewell, Suffolk (http://www.bbc.co.uk/news/business-22772441). Other low carbon technologies like off-shore wind power plants got guaranteed strike prices between 105 £/MWh (in 2015) and 57.50 £/MWh (in 2017) (http://www.offshorewind.biz/2017/09/11/three-offshore-wind-projects-secure-contracts-for-difference-as-strike-prices-go-down/). However, we shouldn’t forget that the strike prices for offshore wind energy are for an intermittent electricity source where the backup would have to be accounted for in addition which will be a task coming up in relevance in the future with increasing electricity production share of based on wind power. Compared to this the strike price for nuclear seems to be a more reliable approximation due to the highly reliable even tailored to suit a market need delivery.

Based on this numbers, dependent on the strike prices currently offered for low carbon energy with the higher end for nuclear energy, electric energy worth between £ 34 to 57 billion can be produced, see discussion above. This calculates to a rough estimation of £ 0.5 to 1 billion/year for selling the electricity produced in the system. This has to be correlated with the avoided cost for secure storage of the plutonium (see the good housekeeping argument in the introduction) for 60 years are in this case not accounted for. Two points have to be considered in this case: first, “President Barack Obama has described the combined 68 tons of plutonium as enough “for about 17,000 nuclear weapons.” [2], thus there is the threat potential which would be eliminated it the Plutonium is burnt. Second, the cost if an alternative route would be chosen. “The two main alternatives to the use of reactor fuel for plutonium disposition are continued storage and direct disposal.” [2]. Continued storage will be costly and in addition there will always be concerns regarding proliferation and misuse. The cost of direct disposal have been given by “an official at the Savannah River Site [who] has estimated that this disposal route costs about $100,000 per kilogram of plutonium” [2]. The proposed route would lead to a waste volume of ~ 1.4 m^3^ per kg of Plutonium [2], Using this numbers on 75 tons of Pu leads to a cost of ~7.5 billion $ and a final disposal volume of more than 100 000 m^3^ which leads to a product still on ground. To provide a better understanding for the required volume, the German high level waste repository programme has been calculated to 30000 m^3^ (http://media.bfs.de/endlagerausstellung/container_1_00.html) disposal volume for 20 power reactors and an averaged reactor operational period of 30 years which accounts to 600 reactor operation years. Thus significant final disposal cost would come extra, but can’t be accounted here, since the fission products and in a conservative analysis, the remaining core composition, would have to be put to a final disposal, too. A rough guess shows that the waste volume of the reactor operation would be less than 5% of the 100000 m^3^ expected for storing 75 tons of Plutonium since it would be 8 tons of heavy metal and the rest would be fission products mostly with a maximum half-life of 30 years compared to 75 tons of Pu. Thus the final disposal cost would be significantly reduced.

### Some safety considerations

The use of pure weapon grade Plutonium in a reactor will cause some safety implications on the kinetic behaviour and the operational stability, mainly due to the reduced delayed neutron fraction produced by the fissile material. This will maybe require some adaption for each reactor system. In the molten salt reactor the initial core composition will maybe require some adoption (e. g. addition of a small amount of depleted Uranium). However, these are considerations which would require a full reactor design which is currently not available especially since the fraction of the fuel outside of the core is here one of the main criterion. The rapid change of the composition as given in Fig 5 and an advanced design will be points to have a detailed look into when the proposal is followed up. The operational stability of a molten salt fast reactor is comparably high due to the coincidence of fuel and coolant which leads in a proper design to a strong negative feedback effect. In a fast reactor system the Pu content in the MOX fuel would have to be adopted slightly in a more detailed study due to the higher criticality resulting from the use of weapon grade Plutonium in the first recycling stage but not in the following stages of a required multi recycling scheme. The reactor kinetics and operational stability could maybe require to have only a partial load based on weapon grade Plutonium. In a light water reactor a full core design would be needed to have detailed safety investigations, specific safety studies would be required due to the higher reactivity provided by the weapon grade plutonium and the influence of the Plutonium on the safety parameters and the kinetic behaviour of the core which could lead to a limitation of the number of MOX fuel assemblies to a certain percentage of the full number of fuel assemblies (e.g. 30% or 50% MOX loading).

## Conclusions

The successful implementation of disarmament treaties of the last centuries has led to significant amounts of weapon-grade Plutonium which are currently stored in high security storage facilities. In the beginning, the requirements for successful Plutonium disposition are discussed as well as the possible disposition routes. In the following, the application of molten salt reactors which have been proposed for highly efficient transmutation and for the burning of civil plutonium is discussed to answer the question: ‘Could these systems be used for the disposition of weapon grade plutonium?’ Possible advantages regarding efficiency, safety and security of the application of liquid fueled reactors are worked out. In a neutronic feasibility study, the Plutonium burning performance of a molten salt fast reactor is compared to the mixed oxide fuel application in a light water reactor and a sodium cooled fast reactor. The molten salt reactor shows a clear advantage in the burning efficiency. In the investigated case the molten salt reactor achieves almost 90% burning efficiency, while the light water reactor achieves ~ 36% efficiency and the fast reactor achieves only a burning rate of 10%.

The investigation on disposal of weapon-grade Plutonium is finished with an analysis of the commercial opportunities of efficient Plutonium burning. The 75 tons of Plutonium which could be burnt in a 3 GW molten salt reactor can lead to electricity production worth between £ 34 to 57 billion depending on the strike prices currently offered for low carbon electric energy. A rough comparison to the alternative approach ‘immobilizing the plutonium with high-level radioactive waste’ is given regarding the required space in a final disposal is given. The disposal via the molten salt reactor route is expected to require only about 5% of the disposal space than the immobilization approach and the cost of ~7.5 billion $ for the immobilization could be avoided.

On the one hand, we should be clear about the significant research and development demand which is required to get a system like the proposed one into operation. On the other hand, the whole topic of weapon-grade Plutonium disposing has not been a real success story up to now. The disruptive approach disposing this Plutonium in a molten salt reactor could become a new, attractive option. It has the potential to be an attractive opportunity avoiding the hassle and eliminating the risk of high security Plutonium storage. In conclusion, burning of the weapon-grade Plutonium resulting from disarmament could be an economically very attractive approach to reduce the nuclear threat.
